# Barriers and facilitators of adherence to medical advice on skin self-examination during melanoma follow-up care

**DOI:** 10.1186/1471-5945-13-3

**Published:** 2013-03-01

**Authors:** Annett Körner, Martin Drapeau, Brett D Thombs, Zeev Rosberger, Beatrice Wang, Manish Khanna, Alan Spatz, Adina Coroiu, Rosalind Garland, Gerald Batist

**Affiliations:** 1 Department of Educational and Counselling Psychology, McGill University, 3700, rue McTavish, Montréal, QC, H3A 1Y2, Canada; 2 Lady Davis Institute for Medical Research, Jewish General Hospital, 4333, Chemin de la Côte-Ste-Catherine, Montréal, QC, H3T 1E4, Canada; 3 Louise-Granofsky-Psychosocial Oncology Program, Jewish General Hospital, 4333, Chemin de la Côte-Ste-Catherine, Montréal, QC, H3T 1E4, Canada; 4 Melanoma Clinic, Royal Victoria Hospital, MGill University Health Centre, 687 Pine Avenue West, Montréal, QC, H3A 1A1, Canada; 5 Department of Dermatology, Jewish General Hospital, 3755, Chemin de la Côte-Ste-Catherine, Montréal, QC, H3S 1X2, Canada; 6 Department of Pathology, Jewish General Hospital, 3755, Chemin de la Côte-Ste-Catherine, Montréal, QC, H3S 1X2, Canada; 7 Segal Cancer Centre, Jewish General Hospital, 3755, Chemin de la Côte-Ste-Catherine, Montréal, QC, H3S 1X2, Canada

**Keywords:** Melanoma, Secondary prevention, Health behavior, Skin self-examination, Medical advice, Distress, Coping, Physician support, Partner support, Skin cancer

## Abstract

**Background:**

Melanoma is the fastest growing tumor of the skin, which disproportionately affects younger and middle-aged adults. As melanomas are visible, recognizable, and highly curable while in early stages, early diagnosis is one of the most effective measures to decrease melanoma-related mortality. Skin self-examination results in earlier detection and removal of the melanoma. Due to the elevated risk of survivors for developing subsequent melanomas, monthly self-exams are strongly recommended as part of follow-up care. Yet, only a minority of high-risk individuals practices systematic and regular self-exams. This can be improved through patient education. However, dermatological education is effective only in about 50% of the cases and little is known about those who do not respond. In the current literature, psychosocial variables like distress, coping with cancer, as well as partner and physician support are widely neglected in relation to the practice of skin self-examination, despite the fact that they have been shown to be essential for other health behaviors and for adherence to medical advice. Moreover, the current body of knowledge is compromised by the inconsistent conceptualization of SSE. The main objective of the current project is to examine psychosocial predictors of skin self-examination using on a rigorous and clinically sound methodology.

**Methods/Design:**

The longitudinal, mixed-method study examines key psychosocial variables related to the acquisition and to the long-term maintenance of skin self-examination in 200 patients with melanoma. Practice of self-exam behaviors is assessed at 3 and 12 months after receiving an educational intervention designed based on best-practice standards. Examined predictors of skin self-exam behaviors include biological sex, perceived self-exam efficacy, distress, partner and physician support, and coping strategies. Qualitative analyses of semi-structured interviews will complement and enlighten the quantitative findings.

**Discussion:**

The identification of short and long-term predictors of skin self-examination and an increased understanding of barriers will allow health care professionals to better address patient difficulties in adhering to this life-saving health behavior. Furthermore, the findings will enable the development and evaluation of evidence-based, comprehensive intervention strategies. Ultimately, these findings could impact a wide range of outreach programs and secondary prevention initiatives for other populations with increased melanoma risk.

## Background

### Melanoma – prevalence, survival and risk factors

Cutaneous melanoma, which disproportionately affects younger and middle-aged adults, is the fastest growing skin tumor. It is the most common form of cancer in women aged 25–29 and is second only to breast cancer in women 30–34 years old. Approximately 60% of all melanomas occur before the age of 65 years [1-5]. Compared to most other cancers, which generally begin to metastasize when they reach a diameter of approximately 1 cm, melanomas can metastasize when they are only 1 mm in depth, which translates into a 1000-fold increase in inherent metastatic potential [6,7]. Hence, the depth of a melanoma at diagnosis is the strongest individual predictor of survival as there are no effective therapies once the tumor has spread [8,9].

Populations with an increased risk for developing melanoma include individuals previously diagnosed with melanoma, first-degree relatives of melanoma survivors, patients with non-melanoma skin cancer, and individuals with many or atypical moles [10-12]. A personal history of melanoma is associated with a life-long elevated risk for developing subsequent melanomas [12-14], with up to 11 of 100 melanoma patients developing a second melanoma, typically within 7 years of the first [15-18].

There is consensus within the clinical and scientific communities that 1) intervention strategies designed to reduce cutaneous melanoma-related mortality must focus on early diagnosis of pre-metastatic tumors [11], p. 50]; and that 2) the highest impact intervention strategies will target high-risk individuals [10-12,19,20]. Because melanoma is recognizable and highly curable when detected early, but increasingly therapy-resistant and lethal as the tumor progresses, secondary prevention interventions can have a significant impact on reducing mortality [12,21-24]. Secondary prevention of melanoma involves early detection via clinical skin exams or skin self-exams [10,25].

### The importance of skin self-examination (SSE)

About 75% of melanomas are detected by patients themselves or by spouses, friends, or other lay persons [26-30]. Early detection programs have been proven successful for the general public and for high-risk populations. For example, a large-scale melanoma-screening program involving participant education on SSE significantly reduced melanoma-related mortality by decreasing the incidence of melanomas with a thickness greater than 0.75 mm [31]. Similarly, an Australian randomized controlled trial (RCT) found that a population screening program, which included a melanoma awareness campaign, led to a reduction in thickness of melanomas diagnosed during the campaign [32,33]. A large-scale population-based case–control study found that SSE was significantly associated with decreased risk of secondary melanoma and of advanced disease, and that SSE reduced melanoma-related mortality by 63% [34]. In this study patients who conducted rigorous SSE, using mirrors to examine one’s back, presented with significantly thinner melanomas than participants who did not perform SSE. A subsequent study with 816 melanoma patients supported the benefits of SSE as early diagnosis was significantly related with the practice of SSE [27]. A prospective study including 2,008 patients diagnosed with stage I - IV melanoma demonstrated that early detection of recurrence results in a significant benefit regarding overall survival probability [19]. Finally, in a study with 1,062 melanoma patients (stages I & II) 19% of patients experienced a melanoma recurrence, which was most often self-detected and led directly to seeking early medical advice [35]. Self-detection, not physician detection, independently predicted survival in this study. Consequently, guidelines from the National Comprehensive Cancer Network (NCCN), an alliance of 21 of the world's leading cancer centers, state that upon completion of the melanoma staging and treatment procedures all patients with stage IA to stage IV melanomas should be advised to self-examine their skin monthly [36]. Dermatological and cancer associations also recommend the regular practice of SSE and provide education materials on SSE conduct for the general public [37-39].

In sum, there is evidence that individuals who perform SSE present for treatment of melanomas at an earlier disease phase, have 50% less advanced melanoma and demonstrate significantly lower melanoma-related mortality [10,27,34,35]. Many melanoma survivors do not, however, practice regular SSE following their initial treatment. Thus, an important challenge is how to achieve better adherence to this practice [40-42].

### Facilitating skin self-examination

Even though cutaneous melanoma are readily visible on the skin surface and it is well-known that SSEs are related to better prognosis [32,40,41,43,44], most melanoma survivors do not perform systematic skin exams regularly [27,34,45,46]. These observations have prompted research efforts to determine factors that may influence whether SSE is performed. Studies have shown, for instance, that having a personal or family history of skin cancer [40] is related to SSE. Demographic characteristics associated with SSE in the general population as well as in melanoma survivors include being female and having a higher level of education [27,40,45]. Unlike medical and demographic factors linked to SSE, which are not generally amenable to intervention, psychosocial and educational factors associated with SSE are potential targets of interventions to improve adherence to SSE instructions. Psychosocial and educational factors associated with SSE behaviors in melanoma survivors and other high-risk individuals include greater knowledge about melanoma and SSE [40,47,48], higher perceived susceptibility [40,49], positive attitude towards SSE [40,50], confidence in being able to perform an efficacious skin self-exam [40,48-50], and being comfortable with having one’s partner assist in SSE [51,52]. There is also preliminary evidence that the level of anxiety and the psychosocial strain resulting from a melanoma diagnosis affect self-exam practice [53]. Furthermore, being informed about SSE by a health care professional has been shown to be associated with SSE performance [45,50,54,55], and several intervention studies in high-risk populations have reported improvement of SSE after standardized dermatological education [50,56,57]. However, improved SSE was documented for only 37% to 63% of the participants of these studies. Longer term effects of the dermatological education could not be determined as the follow-up period only ranged from 3 to 6 months in these studies. Furthermore, little is known about the patients who do not respond to dermatological education. Given that existing interventions to improve SSE have had limited success, it is important to better understand the degree to which patients adhere to SSE and to identify potentially modifiable factors that may influence adherence.

### Limitations in existing evidence on psychosocial factors that may influence skin self-examination

Existing research on the association between psychosocial factors and SSE has been limited by how SSE has been operationalized, the limited inclusion of psychosocial variables, and the duration of studies. Estimates of SSE vary substantially depending on the way the information is elicited [25,58,59]. In some studies, this information has been collected by simply asking patients if they perform SSE without inquiring about the surface of skin examined or by inquiring ambiguously about frequency (e.g., rarely, sometimes, often) instead of using specific frequency categories (e.g., weekly, monthly, twice a year). As a result, rates of thorough SSE amongst melanoma survivors have ranged between 14% and 75% in different studies [30,34,45,46,60,61]. Another limitation of previous research is that key psychological variables, such as psychological distress, coping and professional/personal support, are widely neglected in relation to SSE. First, while distress is a very well known risk factor for non-compliance with medical advice in general [62-64], *only few* studies assessing SSE included a measure of psychological distress [65,66]. Second, *while* research has shown a higher prevalence of avoidant coping in skin cancer patients compared to both other cancer patients and healthy controls [67-72], only one study has examined the potential link between coping strategies and SSE in melanoma survivors [66]. Third, social support plays a crucial role in the psychological adjustment to living with the threat of melanoma [73,74]. Research indicates that social support and coping are strongly interdependent in melanoma patients [69,75]. Research has also shown that physician support is particularly important for melanoma patients, who indicated "trusting my doctors" and "following the medical advice exactly" as the two most frequent coping behaviors right after melanoma removal as well as during follow-up care [53]. Yet, to date only one study has examined coping behaviors in relation to SSE and physician support of SSE hasn’t been studied at all. Furthermore, research has shown that adherence to health behavior recommendation tends to decrease over longer time periods [62,76]. However, previous studies documenting adherence to standardized SSE instructions only involved a 3 to 6-month follow-up assessment [50,56,57]. And lastly, *while* the combination of quantitative and qualitative methods can provide a more complete understanding of a phenomenon, mixed-method studies investigating the barriers and facilitators of SSE are lacking.

In sum, early detection of melanoma via SSE is effective for decreasing melanoma-related mortality. However, despite being recommended by clinical care guidelines, the majority of high-risk individuals do not practice SSE regularly or thoroughly. Rates of SSE can be improved through patient education, but little is known about those who do or do not respond optimally to medical advice on SSE. Acquiring knowledge on the best predictors of SSE practice will enable researchers and clinicians to design intervention protocols to target core issues in melanoma prevention, and, thus, hopefully, reduce mortality rates from secondary melanoma.

### Study objectives

The main objective of this study is to identify short- and long-term predictors of SSE after providing best-practice clinical care, which includes medical advice on SSE, and to better understand challenges and opportunities for secondary prevention of melanoma in high-risk individuals. Specific objectives include:

(1) To determine the extent of thorough SSE performance, defined in terms of completeness and frequency, at 3 months (i.e., Endpoint 1) and at 12 months (i.e., Endpoint 2) after receiving a standardized dermatological education session on SSE during melanoma follow-up care.

(2) To identify psychosocial variables, including distress, coping strategies, and physician and partner support that are independently associated with thorough SSE at 3 months and at 12 months following a standardized dermatological education session.

(3) To use qualitative methods to understand psychosocial factors, including physician and spousal support of SSE (or lack thereof), that help patients to affectively, cognitively and behaviorally adjust to the melanoma diagnosis and to the continuous need for SSE.

### Hypotheses

1. The prevalence of thorough SSE will be higher during the first 3 months than during month 9 to 12 after standardized dermatological education on SSE.

2. Sex will predict SSE in terms of frequency and completeness. Psychosocial variables, including partner and physician support, psychological distress and coping strategies, will significantly predict additional variance in SSE performance assessed at 3 months and at 12 months after standardized dermatological education on SSE.

3. Physician and spousal support of SSE will play an important role for the patient’s psychological adjustment to the melanoma diagnosis and to the continuous need for SSE.

## Method/Design

### Participants and procedures

Ethical approval of the study protocol was granted by the Institutional Review Board of the Faculty of Medicine, McGill University (reference no. A11-B39-11B). Eligible patients will include English- and French-speaking adult patients with a confirmed diagnosis of melanoma who seek services at McGill teaching hospitals. Together, these hospitals treat over 400 new melanoma patients annually [77,78]. Previous psychosocial studies with cancer populations at the same hospitals [30,79,80] and at other sites [81-92] reported participation rates between 57-86%, with a mean of 76%. Attrition rates range between 4% and 24% in psychosocial studies with an assessment up to 18 months after the treatment of patients with melanoma [56,93] or other cancers [67,82,83,94-97]. Enrollment will take place over 2 years and continue until 200 participants have completed the study. A sample of 200 participants provides sufficient statistical power for the analyses (for power estimates see section Data analysis). At all time points the questionnaires will be provided as paper-pencil versions. Participants will be asked up to 3 times to mail back questionnaires completed at home. Interviews will be conducted over the telephone.

During regular clinic visits up to 12 months after their melanoma diagnosis, i.e., at time 1 (T1), eligible patients will be advised by the clinical care staff about the opportunity to participate in the study. In addition, the study will be advertised through flyers and posters in the waiting room area, which will allow interested patients to proactively contact the research team about study participation. The research assistant will provide study information to interested patients, verify study eligibility and collect written, informed consent. Participants will be asked to complete baseline questionnaires on sociodemographic and illness-related information, past SSE behaviors, and psychological functioning at the clinic. Alternatively participants can complete the questionnaires at home and return them to the research team by mail. The research assistant (RA) will access the participants’ medical charts in order to complete the medical information sheet. The second assessment will take place in conjunction with the delivery of the dermatological education at the clinic 3 to 6 months after T1, i.e., at time 2 (T2). Participants will complete questionnaires about distress, coping strategies, physician support and about SSE knowledge, attitude, and self-efficacy. In addition, participants who, at the time of data collection, report having a spouse (i.e., having a committed intimate relationship), will be asked to answer questions related to their partner’s impact on SSE practice. Furthermore, these patients will be asked to take home a survey for their spouse, which mirrors the partner-related questions that the patient is asked to answer regarding SSE. Also at time 2, all study participants will receive a 20-minute standardized dermatological education on SSE derived from empirical evidence [14,29,47,50,65,98-104] and best practice guidelines [36,38,105-109]. Patients will be encouraged to attend the education session together with a significant other (e.g., spouse, other family member, friend) who could assist them with SSE. During the education session, the dermatology-trained RA, i.e., the health educator, will emphasize the usefulness of monthly whole body exams as an effective measure to detect suspicious skin lesions as early as possible. The educator will provide detailed information about how to conduct an effective SSE. This will include an explanation of the well-established ABCDE paradigm (lesion Asymmetry; Border irregularity; Color variation; Diameter; Evolution, e.g. change in size, shape, symptoms, etc.) [110-113] for the detection and interpretation of pigmented lesions by lay persons and health care professionals. Furthermore, the educator will provide handouts, which will assist participants with regular SSE, including a summarizing brochure on melanoma [114], a bookmark with color printed examples of lesions [111], a leaflet providing the link to an online video modeling skin self-examination [115], and a SSE Journal to record skin spots of concern and body parts covered during each home SSE [116,117]. At the end of this 20-minute session, the educator will encourage the participant to reflect on their SSE intentions. Subsequently the participant will be asked to note down in the SSE journal *if*, *when*, *where*, and *assisted by whom* the participant plans to conduct self-exams. At time 3 (T3), i.e., 3 months after the dermatological education, participants are invited by letter to fill out questionnaires assessing distress, coping strategies, physician and spousal support as well as SSE knowledge, attitude, and self-efficacy. In addition, a 10-minute, structured SSE behavior interview with the RA will be scheduled for T3 to assess practice of SSE over the last 3 months. At time 4 (T4), 12 months after the dermatological education, participants will be invited by letter to complete an assessment identical to T3, i.e., questionnaires and SSE behavior interview. At T4 a subsample of 30 patients, which will include the first 15 men and the first 15 women who consent to participate, will be invited to take part in a 50-minute individual, semi-structured interview based on the McGill Illness Narrative Interview [118] focusing on the following questions: 1) how patients deal with the diagnosis and how they experience the illness; 2) their experience regarding SSE, including how they deal with the ongoing need for thorough SSEs and obstacles to SSE; 3) their experience of physician support of SSE, or lack thereof, and how they deal with it; and 4) their experience of partner support of SSE, or lack thereof, and what facilitates or hinders partner support and how they deal with it. Biological sex is considered explicitly given that previous melanoma research suggests that this is a key variable in relation to SSE [27,45,119-121]. Participants will be invited to share and report their thoughts, feelings, and behaviors in each of the 4 domains, which were selected based on their relevance to the cancer trajectory and their presumed significance for SSE practice.

### Measures

#### Descriptive and independent variables: predictors of skin self-examination

Study measures were selected based on their wide use in cancer research, on their psychometric properties, and on their direct relevance to the objectives of the project. They are used to assess descriptive and independent variables, which are expected to have an effect on SSE behaviors during the first 3 months (T1) and 12 months (T2) after dermatological education.

The Sociodemographic Information Form [67] includes questions about age, sex, education, having a spouse, socioeconomic status, and cancer diagnosis. With the Medical Information Sheet [30] the RA gathers data such as time since diagnosis, melanoma stage and depth, previous diagnosis of cancer, melanoma treatment and disease progression. The Skin Cancer Prevention Scale [122] captures whether patients performed SSE and have been advised by health care providers about SSE prior to their current melanoma diagnosis. The Skin Cancer Knowledge Scale, based on questionnaires by Hay et al. 2006 [65] and Manne et al. 2006 [45], assesses knowledge regarding melanoma risk, melanoma warning signs and SSE. The SSE Attitude Scale, an adaptation of Manne’s SSE Benefits and Barriers Scale [45], assesses perceptions of SSE importance, personal gain through SSE, and barriers to SSE. The SSE Self-Efficacy Scale, based on Weinstock et al. 2007 [123], captures an individual’s self-confidence in performing effective skin self-exams. The Physician SSE Support Scale [124] inquires about the perceived interest that the treating physician conveys regarding the patient’s practice of SSE. The Berlin Social Support Scale [125] assesses the patient’s perception of emotional, instrumental and informational illness support provided by their spouse (patient perception and partner perception). The Skin Cancer Index [126,127] is a self-report scale focusing on emotional, social and appearance-related concerns associated with skin cancer. The Patient Health Questionnaire, PHQ-4, [128] screens for symptoms of depression and anxiety. The COPE Inventory [129] inquires about an individual’s use of coping strategies, e.g., mental disengagement, behavioral disengagement, focus on and venting of emotions, use of instrumental social support, denial, humor, use of emotional social support, planning and others.

#### Dependent variables: skin self-examination

Adherence to medical advice on SSE is assessed at 3 months (T3) and at 12 months (T4) after the standardized education session. Based on prior SSE research [30,45,58] and on recommendations for a monthly, comprehensive skin self-exam from the Canadian Dermatology Association [130], the American Academy of Dermatology [108] and the American Cancer Society [38], a structured SSE-behavior interview was developed and pilot tested [30]. During this 10-minute interview the RA records SSE behaviors in terms of SSE completeness and frequency. With the help of a calendar and the body map, which participants receive during the education session in order to document each SSE at home, SSE is recorded for five specified areas of the body: 1) head and neck; 2) front upper body including arms and shoulders; 3) front lower body including groin/genital area, legs, and feet; 4) back upper body including lower back; 5) back lower body including buttocks *and* back of legs. SSE assistance by significant others and the use of melanoma pictures during the self-exams will be documented. The first dependent variable, C*ompleteness of SSE*, refers to the examination of the 5 body areas mentioned above. The RA inquires about each of these 5 parts of the body for each skin self-exam reported by the patient over the last three months at T3 and at T4. The interviewer gives one point for each body area examined, for a possible total of 5 points reflecting the completeness, in regards to the 5 body areas, of each SSE conducted. A mean score (on 5) is calculated across all SSEs. The second dependent variable is *Frequency of SSE* over a given time period. Over the period of 3 months covered by the assessment at T3 and at T4, ideal frequency would be 3 SSEs, with more than 3 SSEs reflecting too high a frequency, and less than 3 reflecting too low a frequency. The total count of SSEs (i.e., how many times a patient performed a SSE) over 3 months will be used to determine if the frequency with which an individual patient conducted SSEs was too low, ideal or too high, which will be used for the data analysis.

### Data analysis

In all relevant cases, additional analyses, including interaction effects and subsample differences, will be examined. **Data analyses related to objective 1**(i.e., determining the extent of thorough SSE performance defined in terms of frequency and completeness): Descriptive and inferential statistics will be employed to gain a differential picture of patients’ practice of SSE in terms of completeness and frequency at T3 and T4 for the total sample as well as for subsamples according to sex, age, having a partner, etc. **Data analyses related to objective 2** (i.e., identifying psychosocial variables independently associated with thorough SSE): First, hierarchical multiple regressions will be performed with SSE completeness as dependent variable using T3 data, i.e., 3 months after standardized dermatological education. The independent variables described above will be entered block-wise, i.e., 1^st^ block: sex; 2^nd^: skin cancer knowledge, attitude toward SEE; 3^rd^: self-efficacy for performing SSE; 4^th^: skin cancer-specific distress measure; 5^th^: partner support and physician support; 6^th^: total score of the coping measure (note that analyses will be repeated with the individual coping subscales). Second, a multinomial regression will be conducted with the same predictor variables and SSE frequency (i.e., too low, ideal and too high frequency) as the dependent variable. The strategies described for T3 will be repeated using the data collected at T4, i.e., 12 months after the education session. With the projected sample size of N = 200, a minimal R^2^ increment of .08 will be detected with .79 power. The power for detecting a R^2^ increment of .09 will be .85. The variance explained by both blocks and individual variables will be examined. The two dependent variables are treated separately because they reflect different aspects of SSE practice. As a second step, they may also be clustered and the resulting composite measure of SSE behavior will serve as the dependent variable in regressions similar to the ones described above. **Data analyses related to objective 3**(using qualitative methods for an in-depth understanding of psychosocial factors related to SSE): Data saturation in qualitative theme analysis, depending on the level of structure of the interview, is often achieved with a sample of 10–15 participants who are examined intensively, while larger samples typically add only minimal new data [131]. Consensual Qualitative Research [131-133] will be applied to the semi-structured interviews of 15 male and 15 female patients. This qualitative method is based on principles of grounded theory [134] and aims at developing a theory directly grounded in the phenomena under study. As a first step, the 2 independent sets of interviews will be theme analyzed to establish preliminary categorizations through open coding. To guide this, the following preliminary questions will be used: how do patients cognitively, affectively and behaviorally adjust to cancer diagnosis and treatment?; how and why do patients engage in or refrain from performing SSE?; how do they experience practicing SSE (e.g., does SSE evoke discomfort, does it trigger tumor fear, does it lead to a sense of safety and control?); in which ways do significant others support or undermine SSE practice?; do patients find it hard to ask significant others for help with SSE or do patients feel overwhelmed by a others’ motivation to help?; and in which ways are physicians experienced as helpful or as non-supportive? Axial coding will then be conducted by which open codes are reviewed and synthesized. As a final step, selective coding will be conducted in order to articulate theories derived from the data and establish the inter-relations between the constructs that were found. By definition, Consensual Qualitative Research is conducted independently by two raters, who then meet to discuss their coding until a consensus is reached. A third independent rater will be available to address disagreements. All proposals are then edited by an editor.

## Discussion

Given the lack of therapeutic options for melanoma, the importance of SSE for the early detection of melanoma, and the limited body of knowledge regarding psychosocial predictors of SSE, this project is expected to generate greatly needed information regarding short- and long-term facilitators and barriers to SSE. By explaining which psychosocial factors affect acquisition and maintenance of SSE, the findings will help to tailor health services and prevention strategies as well as guide professional psychosocial support in order to overcome psychological challenges for sustained SSE practice. The derived knowledge, including information about effective self-management strategies, will not only be disseminated via scientific journals but also amongst health service providers, e.g., within the North-American Cancer Patient Education Network (CPEN), which unites health care professionals to promote models of excellence in patient, family, and community education across the continuum of care. Moreover, the findings are expected to also affect high-risk individuals not previously diagnosed with melanoma by informing secondary prevention efforts for populations such as individuals with non-melanoma skin cancer, with dysplastic nevi or with a family history of skin cancer. Lastly, this study is setting the basis for the design of a state-of-the-art psychosocial intervention program tailored to the SSE risk profile of an individual. The impact of such a psychological complement to standard dermatological education on optimal SSE practice can then be evaluated in an RCT.

## Abbreviations

SSE: Skin self-examination; RCT: Randomized controlled trial; RA: Research assistant; T1: Time 1; T2: Time 2; T3: Time 3; T4: Time 4; FRSQ: Fonds de la Recherche en Santé du Québec; CIHR: Canadian Institutes of Health Research; PORT: Psychosocial Oncology Research Training.

## Competing interests

The authors declare that they have no financial or non-financial competing interests.

## Authors’ contributions

AK conceptualized the study, designed the research protocol, directly oversees the implementation of the study, drafted the research proposal submitted to the funding agency, and wrote the current manuscript. MD contributed substantially to the initial design of the research methodology and performed the power calculations. BT reviewed the initial study design and critically revised the current manuscript for important intellectual content. MD, GB, ZR, and AS contributed to the development of the study protocol, to its implementation at the participating clinical sites and to revisions of the current manuscript. BW and MK supervise the determination of patient eligibility to join the study as well as the delivery of the study intervention. Both provided feedback on the study protocol, on the design of the study intervention and on final revisions of the current manuscript. BW provides the training of the health educator. AC participated in the review and selection of the study measures, designed the study database, oversees data entry, and edited several drafts of this manuscript. RG coordinates the study, contributed to the design and implementation of the study intervention, and provided feedback on this manuscript. All authors read and approved the final manuscript.

## Pre-publication history

The pre-publication history for this paper can be accessed here:

http://www.biomedcentral.com/1471-5945/13/3/prepub
